# Do dental bleaching sessions prior to orthodontic treatment change the bond strength of esthetic brackets?

**DOI:** 10.1590/2177-6709.26.4.e2119124.oar

**Published:** 2021-08-27

**Authors:** Samara Gasperoni PERCIANO, Diego Patrik Alves CARNEIRO, Patricia Rafaela dos SANTOS, Américo Bortolazzo CORRER, Silvia Amélia Scudeler VEDOVELLO, Heloísa Cristina VALDRIGHI

**Affiliations:** 1 Uniararas, Faculdade de Odontologia de Araras, Departamento de Ortodontia (Araras/SP, Brazil).; 2 Universidade Estadual de Campinas, Departamento de Odontologia Restauradora, Divisão de Materiais Dentários (Piracicaba/SP, Brazil).

**Keywords:** Orthodontic brackets, Teeth bleaching, Orthodontics, Shear strength

## Abstract

**Objective:**

The aim of this experimental *in vitro* study was to evaluate whether dental bleaching performed before orthodontic treatment change the shear bond strength (SBS) of monocrystalline and polycrystalline esthetic brackets.

**Methods:**

Sixty (60) bovine incisors teeth were used and randomly divided into the following six groups (n=10): SCP (without bleaching/polycrystalline brackets); SCM (without bleaching/monocrystalline brackets); 1CP (one bleaching session/polycrystalline brackets); 1CM (one bleaching session/monocrystalline brackets); 3CP (three bleaching sessions/polycrystalline brackets); and 3CM (three bleaching sessions/monocrystalline brackets). The brackets were bonded seven days after the bleaching sessions. The samples were submitted to the SBS test in a universal testing machine (Instron model 4411) at 1 mm/min crosshead speed. The two-way analysis of variance (ANOVA) and the Tukey tests were performed at a 5% level of significance. After the mechanical test, samples were evaluated to determine the adhesive remnant index (ARI).

**Results:**

The SBS values were significantly higher for the monocrystalline brackets, when compared with the polycrystalline type (*p*< 0.0001), and significantly higher with three bleaching sessions than without bleaching (*p*< 0.0436). The ARI showed predominance of failures between the bracket and resin for all the groups (score 3).

**Conclusion:**

Three dental bleaching sessions increased the SBS values. Monocrystalline brackets showed higher SBS values than the polycrystalline type.

## RESUMO

**Objetivo:**

O objetivo do presente estudo experimental *in vitro* foi avaliar se sessões de clareamento dental antes do tratamento ortodôntico alteram a resistência da união ao cisalhamento (RUC) de braquetes estéticos dos tipos monocristalino e policristalino.

**Métodos:**

Sessenta (60) dentes incisivos bovinos foram utilizados e divididos aleatoriamente nos seguintes seis grupos (n = 10): SCP (sem clareamento/braquetes policristalinos); SCM (sem clareamento/braquetes monocristalinos); 1CP (uma sessão de clareamento/braquetes policristalinos); 1CM (uma sessão de clareamento/braquetes monocristalinos); 3CP (três sessões de clareamento/braquetes policristalinos); e 3CM (três sessões de clareamento/braquetes monocristalinos). Os braquetes foram colados sete dias após as sessões de clareamento. As amostras foram submetidas ao teste de RUC em máquina universal de ensaios (Instron modelo 4411) na velocidade de 1 mm/min. Foi realizada análise de variância de dois fatores (ANOVA) e teste de Tukey, com nível de significância de 5%. Após o ensaio mecânico, as amostras foram avaliadas para determinação do índice de adesivo remanescente (ARI), de acordo com o método proposto por Artun e Bergland, com o auxílio de uma lupa com lente de aumento de 25x.

**Resultados:**

Os valores de RUC foram significativamente maiores para os braquetes monocristalinos do que para os policristalinos (*p*< 0,0001) e significativamente maiores com três sessões de clareamento do que sem clareamento (*p*< 0,0436). O ARI mostrou predomínio de falhas entre o braquete e a resina para todos os grupos (escore 3).

**Conclusão:**

Três sessões de clareamento dental têm efeito positivo na RUC. Os braquetes monocristalinos apresentaram valores de RUC superiores aos do tipo policristalino.

## INTRODUCTION

Dental bleaching is an easy and conservative therapy for whiting teeth.^1^ ^,^ ^2^ Dental bleaching can be performed at-home or in-office,^3^ ^-^ ^5^ and the most common used substances are carbamide and hydrogen peroxide in various concentrations.^3^ ^,^ ^4^

Patients willing to have white and aligned teeth have associated dental bleaching with orthodontic treatment,^6^ undergoing the procedure before or after orthodontic treatment.^7^

 Adhesive used for bracket bonding may undergo degradation or solubility due to the presence of bleaching agents.^8^ Various methods have been proposed to avoid problems related to reduction in bond strength after bleaching, such as application of antioxidant, desensitizing agents,^2^ ^,^ ^4^ sodium ascorbate, tocopherol acetate, retinol acetate,^9^ or increase post-bleaching time interval for bracket bonding.^1^ ^,^ ^5^ ^,^ ^8^

Patients who are concerned about esthetics during orthodontic treatment have the option of using monocrystalline or polycrystalline esthetic brackets.^10^ Both types are composed of aluminum oxide,^11^ ^,^ ^12^ with different manufacturing process. Polycrystalline ceramic brackets are composed of polycrystalline alumina, made up of aluminum oxide crystals fused at high temperatures, allowing various brackets to be molded simultaneously. Polycrystalline brackets are more common and popular, due to the quality of the material and the relative ease of production. In turn, monocrystalline ceramic brackets consist of a mass cast at a high temperature (2,100^o^C) forming a single aluminum oxide crystal that will result in the fabrication of a single bracket, thus making the production process more expensive. However, this form of milling presents lower incorporation of impurities, making the brackets stronger and more translucent. This difference may favor the transmission of light and influence the polymerization of the resin used for bonding the brackets, therefore generating an influence on the shear bond strength (SBS).^13^

Concern is related to the SBS of brackets bonded after bleaching procedures,^6^ ^-^ ^9^ ^,^ ^14^ and few studies have compared the retention of different types of esthetic brackets used nowadays. Based on the literature, studies of SBS after bleaching treatments refer to a single session of dental bleaching.^1-4,6,8,9,15^ In an endeavor to approximate clinical reality, in which the dentist commonly performs more bleaching sessions, the proposal of the present study was to test the following hypothesis: 1) the number of bleaching sessions performed prior to esthetic bracket bonding may influence the SBS, bearing in mind that bleaching sessions may change the enamel surface, thereby reducing the bond strength; 2) the type of esthetic bracket (monocrystalline or polycrystalline) may influence the SBS. Therefore, the aim was to evaluate, *in vitro*, the SBS of esthetic brackets (monocrystalline or polycrystalline) after one and three dental bleaching sessions. 

## MATERIAL AND METHODS

The present experimental *in vitro* study was approved by the Research Ethics Committee (number 596/2018, *Fundação Hermínio Ometto*). Sixty (60) bovine incisors teeth without fractures, cracks or white stain lesions were used. Freshly extracted teeth without any previous chemical substance treatment were selected for testing. The teeth were cleaned with a periodontal curette, then rinsed and stored in distilled water at room temperature of 37°C^4^. The specimens were thus inserted into PVC tubes and fixed with acrylic resin (JET, São Paulo, SP, Brazil). Teeth were manually positioned so that the vestibular surfaces were set perpendicular to the ground with the aid of an acrylic plate. After the resin cure, prophylaxis of the specimen was performed with a pumice stone and water, with the aid of a Robinson brush, and subsequently the specimens were stored at room temperature.

The specimens were randomly divided into six groups (n = 10). Polycrystalline Roth brackets (Iceram, Orthometric, Marília, São Paulo, Brazil) and monocrystalline Roth brackets (Iceram S, Orthometric, Marília, São Paulo, Brazil) were used.

Groups 1CP and 1CM received dental bleaching, prior to bracket bonding, with 37% carbamide peroxide gel (Office power bleaching 37%, BM4, Palhoça, Santa Catarina, Brazil), for 45 minutes, in accordance with the manufacturer’s instructions. After bleaching, teeth were washed with water/air for 30 seconds and stored at room temperature. Polycrystalline brackets were bonded to Group 1CP; and monocrystalline brackets, to Group 1CM, seven days after the bleaching session.

Groups 3CP and 3CM received dental bleaching in the same way as was performed in Groups 1CP and 1CM; however, three bleaching sessions were performed with a time interval of seven days between each session. Polycrystalline brackets were bonded to Group 3CP; and monocrystalline brackets, to Group 3CM, seven days after the last bleaching session.

For bracket bonding, 37% phosphoric acid (Condac, FGM, Joinville, Santa Catarina, Brazil) was applied for 15 seconds, followed by washing with water jet for 30 seconds and drying with air jet for 15 seconds. Subsequently, Transbond XT Primer adhesive (3M Unitek, Monrovia, USA) was applied on the tooth surface and polymerized for 15 seconds. Transbond XT was applied at the straight wire incisor bracket base (Orthometric). The brackets were manually placed on the tooth surface and the excess was removed with an exploratory probe. Polymerization was performed for 10 seconds on each surface (distal, mesial, cervical and occlusal) with an Optilight Max 440 light polymerization device (Gnatus^®^, wavelength 420 to 480 nm, irradiance 1200 mV/cm^2^) and stored at room temperature until the tests were performed. The brackets were submitted to the SBS test in a universal test machine (Model 4411; Instron Inc., Canton, MA, USA) at a compressive speed of 1 mm/min. The maximum debonding force was divided by the area of the brackets base, and the results were reported in megapascal (MPa). A descriptive analysis of data was performed, and the results were presented as mean and standard deviation. The normality and homoscedasticity of the data were assessed. The results were submitted to two-way analysis of variance, and if difference was observed between the groups, the complementary Tukey’s test was performed (*p*< 0.05).

After the SBS test, the adhesive remnant Index (ARI) was evaluated with the aid of a magnifying glass, with 25x magnification lens, in accordance with the method proposed by Artun and Bergland^16^. Scores ranged from 0 to 3:

0 - Absence of any adhesive remnant layer on the enamel.
1 - Presence of less than half of the resin remnant on the enamel.
2 - Presence of more than half of the resin remnant on the enamel.
3 - Presence of all the resin on the enamel, together with the impression of the bracket base mesh.

## RESULTS

Table 1 shows that the bond strength was significantly higher for the monocrystalline brackets (54.22 ±13.15 MPa) than for the polycrystalline type (20.28 ±11.23 MPa) (*p*< 0.0001). Regardless of the type of bracket, the bond strength was significantly higher with three bleaching sessions (40.30±17.96 MPa) when compared to the control group (32.08±20.93 MPa) (*p*< 0.0436). 

**Table 1:** Mean shear bond strength (MPa) and standard deviation, considering number of bleaching sessions and type of esthetic bracket.

Treatment	Brackets		Tukey
	Polycrystalline	Monocrystalline	
Without bleaching	14.37 (7.80)	49.79 (12.90)	(32.08±20.93)^b^
1 bleaching session	20.04 (10.51)	58.71 (15.70)	(39.38±23.72)^ab^
3 bleaching sessions	26.44 (12.40)	54.16 (10.01)	(40.30±17.96)^a^
Tukey	(20.28 ±11.23)^B^	(54.22 ±13.15)^A^	-

Groups with different superscript letters (capital letters comparing on the horizontal line and lower cases in the vertical differed among them (p ≤ 0.05). p (bracket) < 0.0001; p (treatment) = 0.0436; p (bracket x treatment) = 0.2346.

Evaluation of the ARI demonstrated predominance of score 3 (58/60 - 96,7%), except for Groups 1CP and 1CM, in which one of the test specimens presented a score 2 (2/60 - 3,3%) (Table 2).

**Table 2:** Adhesive Remnant Index of monocrystalline and polycrystalline brackets submitted to 1 and 3 bleaching sessions.

	SCP	SCM	1CP	1CM	3CP	3CM	Total
0	0%	0%	0%	0%	0%	0%	0%
1	0%	0%	0%	0%	0%	0%	0%
2	0%	0%	10% (1)	10% (1)	0%	0%	3.3% (2)
3	100% (10)	100% (10)	90% (9)	90% (9)	100% (10)	100% (10)	96.7% (58)
Total	100% (10)	100% (10)	100% (10)	100% (10)	100% (10)	100% (10)	100% (60)

SCP: without bleaching/polycrystalline brackets; SCM: without bleaching/monocrystalline brackets; 1CP: one bleaching session/polycrystalline brackets; 1CM: one bleaching session/monocrystalline brackets; 3CP: three bleaching sessions/polycrystalline brackets; 3CM: three bleaching sessions/monocrystalline brackets.

## DISCUSSION

The initial hypothesis of this study, that one and three in-office bleaching sessions would reduce the bond strength, was rejected (1); the hypothesis that the type of bracket, monocrystalline or polycrystalline, would influence the SBS was accepted (2).

The present results demonstrated that three bleaching sessions increased the SBS of both types of brackets (monocrystalline and polycrystalline), corroborating the findings of other studies that verified higher SBS in previously bleached groups, when compared to without previous bleaching.^4^ ^,^ ^9^ On the other hand, the study of Andrighetto et al.^2^ observed no differences in the SBS values in brackets bonded after bleaching or not bleached. Moreover, it was also possible to find reports contradicting the present findings, in which the SBS of brackets was higher in the groups without bleaching prior to bracket bonding.^1^ ^,^ ^3^ ^,^ ^15^

It is believed that the differences in the present results, when compared with the literature, was due to the methodological differences, because studies normally compare the group without bleaching and groups submitted to a single dental bleaching session. However, the present research intended to approximate the clinical reality, in which in-office dental bleaching is commonly performed in more than one session, to attain the expected result. Previous studies that compared the surface roughness after dental bleaching have suggested that bleaching promoted changes in the superficial layers of enamel.^17^ ^-^ ^20^ Roughness then justified the SBS increase in the groups exposed to three bleaching sessions, suggesting that more bleaching sessions promoted irregularities in the enamel, thus increasing the mechanical retention of resin on the tooth surface.

When the types of esthetic brackets (monocrystalline and polycrystalline) used in this study were compared, regardless of the condition, the monocrystalline brackets showed higher SBS values. Corroborating these findings, other studies have also observed higher SBS values in monocrystalline brackets, when compared to polycrystalline.^21^ ^,^ ^22^ On the other hand, other researches have found no significant differences between monocrystalline and polycrystalline esthetic brackets.^12^ ^,^ ^23^ The higher SBS values found in the monocrystalline brackets, when compared with the polycrystalline type, may be explained by their composition and manufacturing process, because they have less incorporation of impurities, which makes the bracket more translucent, allowing the bracket to retain less light, directly interfering in the efficiency of resin polymerization, thereby increasing retention to the tooth.^11^ ^,^ ^13^ ^,^ ^22^ ^,^ ^23^

Regarding ARI, score 3 was predominant in the evaluated groups. Ten percent of the sample in Groups 1CP and 1CM presented score 2. Bleaching prior to bracket bonding did not interfere in the ARI, demonstrating high effectiveness of the selected resin for bracket bonding purpose. The present results corroborate the findings of previous studies.^12^ ^,^ ^21^ ^-^ ^23^ This finding is advantageous, because the ideal is to remain all the material on the tooth surface, thus protecting the enamel.^24^ ^,^ ^25^

Finally, the authors suggest that new clinical studies should be carried out with the objective of evaluating the long-term retention of esthetic brackets after dental bleaching, since the results of this study express an *in vitro* setting. 

## CONCLUSION

After three sessions, dental bleaching increased shear strength of aesthetic brackets. The monocrystalline brackets presented the highest SBS values. 
